# Plethysmography as a Complementary Tool for Monitoring Response to CFTR Modulator Therapy in Children with Cystic Fibrosis: A Real-World Study

**DOI:** 10.3390/jcm15218356

**Published:** 2026-10-27

**Authors:** Magdalena Aleksandra Postek, Katarzyna Walicka-Serzysko, Urszula Borawska-Kowalczyk, Justyna Milczewska, Katarzyna Zybert, Anna Wołkowicz, Łukasz Woźniacki, Dorota Sands

**Affiliations:** 1 Cystic Fibrosis Department, Institute of Mother and Child, 01-211 Warsaw, Poland; katarzyna.walicka@imid.med.pl (K.W.-S.);; 2 Cystic Fibrosis Centre, Paediatric Hospital, 05-092 Dziekanów Leśny, Poland

**Keywords:** plethysmography, CFTR modulator therapy, cystic fibrosis therapy, air trapping

## Abstract

**Background**: Sensitive tools for monitoring response to cystic fibrosis transmembrane conductance regulator (CFTR) modulator therapy remain needed in children with cystic fibrosis (CF). Plethysmography may provide complementary information to spirometry and multiple-breath nitrogen washout (MBNW). **Aim:** To characterize longitudinal changes in plethysmographic parameters before and during CFTR modulator therapy in children and adolescents with CF, using spirometry and MBNW as complementary physiological assessments. **Methods**: This retrospective observational study included 67 children with CF. Forty-nine patients received elexacaftor/tezacaftor/ivacaftor (ETI) and 18 received tezacaftor/ivacaftor (TI). Spirometry, plethysmography, and MBNW were performed at baseline and at follow-up, typically 12 months after treatment initiation, but no later than 24 months. Health-related quality of life was assessed using the Cystic Fibrosis Questionnaire–Revised (CFQ-R) in 42 ETI-treated patients with complete paired data. Pulmonary exacerbations requiring intravenous antibiotic treatment were also analyzed. **Results**: During ETI treatment, significant within-group changes were observed in forced expiratory volume in 1 s (FEV1), forced vital capacity (FVC), lung clearance index (LCI_2.5_), airway resistance, total lung capacity (TLC), and the residual volume-to-total lung capacity ratio (RV/TLC). Improvements in selected CFQ-R domains and a reduction in pulmonary exacerbations requiring intravenous antibiotics were also observed in the ETI group, although the between-group difference in exacerbation changes was not statistically significant. **Conclusions:** Plethysmography, particularly airway resistance and RV/TLC, may provide complementary information to spirometry and MBNW when monitoring pulmonary function changes during CFTR modulator therapy in pediatric CF.

## 1. Introduction

Cystic fibrosis (CF) is an autosomal recessive disease caused by pathogenic variants in the gene encoding the cystic fibrosis transmembrane conductance regulator (CFTR), an epithelial ion channel responsible for chloride and bicarbonate transport across cell membranes. Dysfunction of the CFTR protein leads to dehydration of the airway surface liquid, impaired mucociliary clearance, and accumulation of viscous airway secretions. These abnormalities promote chronic infection, persistent neutrophilic inflammation, and progressive structural lung damage. Despite major advances in multidisciplinary care, pulmonary disease remains the leading cause of morbidity and mortality in individuals with CF [1,2].

Over the past decade, the therapeutic landscape of CF has been transformed by CFTR modulator (mCFTR) therapies targeting the underlying molecular defect. These therapies utilize small molecules to improve CFTR protein processing, trafficking, and channel gating, thereby partially restoring epithelial ion transport. Highly effective triple-combination therapy with elexacaftor, tezacaftor, and ivacaftor (ETI) has demonstrated substantial improvements in lung function, nutritional status, and quality of life in adolescents and adults with CF carrying at least one F508del allele [3,4]. Subsequent prospective and real-world studies confirmed the clinical effectiveness of ETI across broader populations, including younger age groups [5,6,7,8]. These therapies are increasingly initiated in pediatric patients, in whom early treatment may alter the course of CF lung disease and potentially prevent irreversible structural damage [9,10].

Monitoring response to CFTR modulator therapy in children remains challenging. Forced expiratory volume in the first second (FEV1), the most commonly used spirometric parameter, is relatively insensitive to early lung disease and may remain within the normal range despite significant structural and functional abnormalities [11]. This limitation is particularly relevant in pediatric populations, where pathological changes frequently begin in the small airways and may progress before measurable spirometric decline occurs. Consequently, increasing attention has been directed toward physiological techniques capable of detecting early abnormalities in ventilation distribution and lung mechanics [12,13,14].

Air trapping is an important pathophysiological feature of cystic fibrosis lung disease and is associated with small-airway obstruction resulting from mucus accumulation, inflammation, and airway wall remodeling [1,2]. These processes lead to incomplete lung emptying during expiration and progressive hyperinflation. Air trapping can be quantified using static lung volume measurements, including residual volume (RV), functional residual capacity (FRC), total lung capacity (TLC), and the RV/TLC ratio. Plethysmography is considered a reference method for assessing lung volumes and airway resistance, allowing detection of hyperinflation and trapped gas even when spirometry results remain unchanged [15,16]. Plethysmographic measurements therefore provide important information about the mechanical consequences of small-airway obstruction in CF.

In recent years, multiple-breath nitrogen washout (MBNW) has gained increasing attention as a sensitive tool for detecting early ventilation inhomogeneity in CF. The lung clearance index at 2.5% of the starting tracer gas concentration (LCI_2.5_), derived from MBNW testing, reflects the efficiency of gas mixing within the lungs and has been shown to be more sensitive than spirometry for identifying early CF lung disease [12,17,18,19]. LCI_2.5_ abnormalities can precede spirometric decline and have been associated with structural lung damage detected by imaging techniques [19,20,21,22]. Several studies have demonstrated improvements in LCI_2.5_ following initiation of highly effective CFTR modulator therapy, suggesting partial restoration of ventilation homogeneity [6,7,8].

Plethysmography and MBNW capture different physiological aspects of CF lung disease. While MBNW primarily reflects ventilation distribution and small airway dysfunction, plethysmography directly quantifies lung volumes, airway resistance, and the degree of hyperinflation and air trapping [12,15,16]. These methods should therefore be considered complementary rather than interchangeable. In addition, plethysmography is widely available in routine pulmonary function tests and may provide clinically relevant information regarding the mechanical consequences of small airway obstruction during follow-up of patients receiving CFTR modulator therapy. Interpretation of plethysmographic parameters requires consideration of their interrelationships, as changes in RV/TLC may reflect alterations in RV, TLC, or both. Therefore, evaluating airway resistance and lung volumes together with spirometric indices and LCI_2.5_ may provide a more comprehensive physiological characterization of lung function during CFTR modulator therapy, although the additional clinical value of this approach remains to be established [23,24,25].

Changes in spirometric indices and ventilation inhomogeneity during CFTR modulator therapy have been extensively investigated [6,7,8]. However, evidence regarding longitudinal changes in plethysmographic parameters in children and adolescents with cystic fibrosis remains limited [24,25]. Although recent studies have reported changes in lung volumes and indices related to air trapping during ETI therapy, data describing changes in airway resistance and static lung volumes in pediatric populations remain scarce [24,25]. The present study contributes to the existing evidence by characterizing changes in plethysmographic parameters before and during CFTR modulator therapy in a real-world pediatric cohort, with spirometry and multiple-breath nitrogen washout providing complementary assessments of pulmonary function.

The primary aim of this study was to assess changes in plethysmographic parameters, including airway resistance, lung volumes, and the RV/TLC ratio, in children and adolescents with cystic fibrosis before and during CFTR modulator therapy. Secondary analyses evaluated changes in spirometric indices and lung clearance index, while health-related quality of life and pulmonary exacerbation rates were assessed as exploratory clinical outcomes. These analyses were intended to characterize changes observed during treatment rather than establish causal treatment effects or compare the clinical superiority of individual pulmonary function testing methods.

## 2. Results

A total of 174 plethysmographic test results were reviewed, and 134 met the predefined validity criteria. Additionally, 94 CFQ-R questionnaires were collected. The comparative analysis categorized by modulator therapy type included 67 patients who met the inclusion criteria and had complete clinical data available, representing approximately 70% of the initial cohort. Among them, 49 patients were treated with ETI and 18 with TI. The characteristics of the study population are presented in Table 1.

Patients in the ETI group were older at treatment initiation than those in the TI group. The mean age was 14.01 ± 1.73 years in the ETI group and 9.72 ± 2.28 years in the TI group. This distribution reflected the availability and eligibility criteria for CFTR modulator therapy at that time. In the ETI group, females constituted 59.2% of patients, whereas in the TI group males predominated, accounting for 61.1% of patients.

The patients carried the F508del variant in a homozygous state or in combination with another variant. Patients who were heterozygous without documented carriage of the F508del variant were not included in the study. The most common genotype in the overall cohort was F508del/F508del, observed in 38 patients (56.7%), 25 in the ETI group and 13 in the TI group. Among heterozygous genotypes, the most frequent was F508del/3849+10kbC>T, present in five patients (7.5%), all of whom were treated with TI. Other heterozygous genotypes were less frequent and were observed only in the ETI group.

Changes in pulmonary function parameters from baseline to follow-up in the ETI group are presented in Table 2.

In the ETI group, significant reductions in airway resistance (Rtot and Reff) and RV/TLC were observed, accompanied by a significant increase in TLC. Significant improvements were also observed in FEV1, FVC, and LCI_2.5_. No statistically significant changes were detected in FRC or RV.

Changes in pulmonary function parameters from baseline to follow-up in TI group are presented in Table 3.

In the TI group, a statistically significant reduction in FVC (% predicted and z-score) was observed, whereas no significant changes were detected in FEV_1_, LCI_2.5_, or any of the plethysmographic parameters.

The distribution of patients exceeding predefined abnormality thresholds is presented in Table 4.

The number of patients exceeding predefined thresholds decreased for several pulmonary function parameters in the ETI group, although the changes were not consistent across all parameters (Table 4). Paired categorical analyses demonstrated statistically significant changes in LCI_2.5_ and FEV1 z-score after adjustment for multiple comparisons. The findings for Reff % predicted were dependent on the testing approach, as detailed in Table 5. No statistically significant categorical changes were detected in the TI group.

After adjustment for multiple comparisons, significant categorical changes in LCI_2.5_ and FEV1 z-score were observed in the ETI group. For Reff % predicted, statistical significance was dependent on the testing approach and was not consistently maintained across the adjusted analyses. Therefore, this finding should be interpreted with caution. No statistically significant categorical changes were detected in the TI group (Table 5).

In the ETI group (*n* = 49), the mean annualized rate of pulmonary exacerbations requiring intravenous antibiotic therapy decreased from 0.59 ± 0.82 to 0.16 ± 0.43 events per patient-year, with a mean within-patient change of −0.43 ± 0.78 events per patient-year (Table 6).

Among the 42 patients with complete paired CFQ-R data, significant improvements were observed in the physical, social functioning, respiratory, and digestive domains after Benjamini–Hochberg adjustment. The largest mean increase was observed in the respiratory domain (12.57 ± 16.11 points; 95% CI: 7.55–17.58; adjusted *p* < 0.001). No significant changes were detected in the remaining domains (Table 7).

Exploratory analyses revealed an inverse correlation between changes in LCI_2.5_ and changes in the CFQ-R respiratory domain score (*n* = 42; Spearman’s ρ = −0.387; 95% CI: −0.616 to −0.090; unadjusted *p* = 0.011). However, this association did not remain statistically significant after Benjamini–Hochberg correction (adjusted *p* = 0.457).

## 3. Discussion

The present study demonstrates that ETI therapy was associated with broad improvement in respiratory physiology in children and adolescents with CF. Importantly, the observed effects were not limited to conventional spirometric indices but also involved parameters obtained from plethysmography, including airway resistance and indices related to lung volumes, hyperinflation, and air trapping. This is clinically relevant because spirometry alone may underestimate peripheral airway disease, especially in pediatric patients with preserved or near-normal FEV_1%_pred. Plethysmography complements spirometry by enabling assessment of static lung volumes and airway resistance, which are essential for identifying obstructive physiology, hyperinflation, and trapped gas [15,16,17,23].

The improvement in FEV_1_ observed in the ETI group is consistent with previous pivotal and real-world studies of highly effective mCFTR therapy. In the phase III trial by Middleton et al., ETI produced substantial improvement in percent predicted FEV1 in patients with at least one F508del allele [3]. Subsequent prospective and observational studies confirmed that ETI improves lung function, reduces respiratory symptoms, and may modify the trajectory of lung disease progression [5,6,7,8]. Our findings are also consistent with pediatric data showing that ETI improves pulmonary function and quality-of-life outcomes in routine clinical practice [6,7,8]. However, the main advantage of the present study is the demonstration that treatment response can also be detected by plethysmographic indices, which provide information beyond airflow limitation alone. Given the exploratory nature of the study and the number of statistical comparisons performed, the findings should be interpreted cautiously. Although the Benjamini–Hochberg procedure was applied to control the false discovery rate, the possibility of false-positive findings cannot be entirely excluded.

In the ETI group, significant reductions in R_tot_ and R_eff_ were observed after treatment. These findings suggest improved airway mechanics and reduced airflow obstruction. In CF, increased airway resistance may result from mucus retention, airway wall thickening, bronchial narrowing, and premature small airway closure during expiration [1,2]. Therefore, the reduction in resistance parameters after ETI treatment may reflect improved airway patency, enhanced mucociliary clearance, and more effective expiratory emptying. Similar observations have recently been reported in studies specifically evaluating body plethysmography after ETI, in which triple CFTR modulator therapy improved not only FEV1 but also airway resistance and hyperinflation-related parameters [25].

A notable finding of this study was the significant reduction in RV/TLC during ETI treatment, observed both in percent predicted values and z-scores. RV/TLC represents the proportion of total lung capacity remaining after maximal expiration and is commonly used as an indicator of air trapping. However, because RV/TLC is influenced by changes in both RV and TLC, a reduction in this ratio does not necessarily indicate a decrease in absolute residual volume [23,25].

In obstructive lung disease, early closure of peripheral airways during expiration leads to increased RV and increased RV/TLC, indicating air trapping and resting hyperinflation [15,16,17]. Thus, the reduction in RV/TLC observed in our cohort suggests a decrease in the relative burden of trapped air during ETI therapy.

The interpretation of changes in TLC and RV/TLC requires caution. In the ETI group, TLC increased significantly, while RV/TLC and airway resistance decreased. These findings may reflect changes in pulmonary function during treatment; however, their physiological significance remains uncertain. As RV did not change significantly, the reduction in RV/TLC may have been influenced, at least in part, by the increase in TLC. Further studies are needed to clarify the clinical relevance of these observations.

This interpretation is supported by recent studies evaluating plethysmographic outcomes following ETI therapy. Gramegna et al. demonstrated that after 12 months of treatment in patients with advanced cystic fibrosis lung disease, ETI significantly reduced RV/TLC and FRC/TLC while improving specific airway resistance. Moreover, they showed that the degree of baseline hyperinflation predicted the magnitude of FEV1 improvement, suggesting that lung volume abnormalities contribute to the heterogeneity of treatment response and may have prognostic value [25]. Complementing these findings, Colin et al. reported that despite substantial improvements in lung function after ETI, a proportion of patients continued to exhibit persistent lung hyperinflation and small airway dysfunction. This observation indicates that residual physiological abnormalities may remain even when spirometric parameters improve, highlighting the added value of body plethysmography in detecting changes that are not fully captured by FEV1 alone [24,25]. Together, these studies support the use of plethysmographic assessment as a complementary tool for monitoring the response to CFTR modulator therapy, providing clinically relevant information on hyperinflation and airway mechanics beyond conventional spirometry.

The relevance of air trapping is especially important in pediatric CF. Small-airway disease may precede overt spirometric impairment, and children may present with substantial ventilation inhomogeneity or trapped gas despite normal or near-normal FEV1 [18,19]. Air trapping reflects small-airway obstruction caused by mucus plugging, inflammation, and airway wall remodeling. Functional and imaging studies have shown that air trapping and ventilation abnormalities may be among the earliest detectable manifestations of CF lung disease [19,20,26]. In children and adolescents with CF, MRI-based studies have shown that pulmonary function markers of ventilation inhomogeneity and air trapping correlate with perfusion abnormalities, supporting their role as indirect markers of peripheral airway involvement [26].

The significant improvement in LCI observed in the ETI group further supports the hypothesis that ETI improves peripheral airway function and ventilation distribution. LCI_2.5_ derived from MBNW testing is increasingly recognized as a sensitive marker of early CF lung disease and small-airway dysfunction [12,13]. It may detect treatment effects even when spirometry remains within the normal range. Previous studies have demonstrated that LCI correlates with structural lung disease on imaging and may be useful as an outcome measure in clinical trials and real-world monitoring [19,21,22]. In randomized pediatric studies, ETI significantly reduced LCI_2.5_ and improved FEV 1% predicted and CFQ-R respiratory domain scores [6]. Real-world pediatric data have also confirmed clinically meaningful improvements in LCI_2.5_ and respiratory outcomes after ETI initiation, although the magnitude of LCI_2.5_ change may depend on baseline disease severity and lung health [7,8].

In the present study, an exploratory analysis revealed a moderate inverse association between changes in LCI_2.5_ and changes in the CFQ-R respiratory domain scores. Although this association was nominally significant, it did not remain statistically significant after correction for multiple comparisons. Therefore, the finding should be interpreted cautiously and considered hypothesis-generating rather than confirmatory. Nevertheless, MBNW and plethysmography provide complementary physiological information: LCI_2.5_ reflects ventilation inhomogeneity, whereas plethysmography characterizes airway resistance and static lung volumes, including indices related to air trapping.

Improvements in selected CFQ-R domains were observed alongside changes in pulmonary function parameters during ETI treatment. Statistically significant improvements were found in the physical, social functioning, respiratory, and digestive domains after adjustment for multiple comparisons. These findings are consistent with previous studies reporting both improvements in pulmonary function and patient-reported outcomes during ETI therapy [3,4,5,6,7,8].

In the TI group, no statistically significant changes were observed in most pulmonary function parameters, including airway resistance and lung volumes. Previous studies have reported improvements in LCI_2.5_ during TI treatment in children with eligible CFTR genotypes [10]. Furthermore, a randomized clinical trial directly comparing ETI with TI demonstrated greater improvements in selected clinical outcomes with ETI [9]. However, the present study was not designed to assess comparative treatment efficacy, and the observed within-group changes should not be interpreted as evidence of differences between the two regimens.

Another clinically relevant observation was the reduction in intravenous antibiotic-treated pulmonary exacerbations after ETI therapy. A reduction in the annualized rate of pulmonary exacerbations requiring intravenous antibiotic treatment was observed in the ETI group. This finding is consistent with previous clinical trials and observational studies reporting reductions in pulmonary exacerbations during ETI treatment [3,5]. However, given the retrospective design of the present study, this observation should be interpreted as an association rather than evidence of a causal treatment effect.

Overall, the findings suggest that body plethysmography may provide complementary information on pulmonary function during CFTR modulator therapy, alongside spirometry and LCI_2.5_. However, the additional clinical value of these measurements requires further investigation.

### Study Limitations

The present study has several limitations. Its retrospective observational design precludes causal inference, while the relatively small and unequal treatment groups and differences in age, sex distribution, and CFTR genotype limit direct comparisons between ETI and TI. Accordingly, the findings describe within-group changes during treatment rather than differences in comparative treatment efficacy.

Follow-up intervals varied between 12 and 24 months, and growth and maturation may have influenced lung volume measurements despite the use of age-adjusted reference values. In addition, approximately 30% of initially screened patients were not included in the final analysis because follow-up data were unavailable, partly due to the transition from pediatric to adult CF care. Potential selection bias therefore cannot be excluded.

Plethysmographic findings also require cautious interpretation. In the ETI group, the reduction in RV/TLC was accompanied by an increase in TLC, while the change in RV was not statistically significant. Consequently, the observed reduction in RV/TLC cannot be taken as direct evidence of a reduction in absolute residual volume or trapped gas. The absence of corresponding imaging data further limited the assessment of potential changes in regional ventilation and structural lung abnormalities.

Additional limitations concern the reference equations and classification thresholds used in the plethysmographic analyses. Reference values were based on the equations of Zapletal et al. (1987) [27], although newer GLI-2021 reference equations are available for static lung volumes [28]. For Rtot and Reff, values > 120% predicted were classified as elevated using a study-defined threshold rather than a universally validated upper limit of normal. Furthermore, parameter-specific criteria were applied in the categorical analyses: RV and TLC z-scores were classified according to elevated values only, whereas RV/TLC z-scores outside the range of −1.64 to +1.64 were considered abnormal. These analyses therefore assessed transitions between predefined categories rather than the full spectrum of physiological abnormalities.

The relatively small sample size and multiple statistical comparisons also increase the uncertainty of individual findings. Although the Benjamini–Hochberg procedure was applied to address multiple testing, the possibility of false-positive findings cannot be excluded. The exploratory correlation between changes in LCI_2.5_ and CFQ-R respiratory domain scores did not remain statistically significant after adjustment for multiple comparisons. Moreover, established thresholds for clinically meaningful changes in airway resistance, TLC, and RV/TLC during CFTR modulator therapy in children are lacking. Therefore, statistically significant changes in these parameters should not automatically be interpreted as clinically meaningful improvements.

Overall, the findings should be considered exploratory and hypothesis-generating. Further prospective studies with standardized follow-up intervals, contemporary reference equations, imaging assessments, and relevant clinical outcomes are needed to clarify the clinical significance of plethysmographic changes during CFTR modulator therapy.

## 4. Materials and Methods

### 4.1. Patients and Study Design

This retrospective observational study included children and adolescents aged 6–18 years with cystic fibrosis who were eligible for CFTR modulator therapy according to their genotype and national reimbursement criteria. Treatment was initiated in 2022. Of the 97 patients initially screened, 67 had the required clinical and pulmonary function data available at baseline and follow-up and were included in the final analysis (49 receiving elexacaftor/tezacaftor/ivacaftor (ETI) and 18 receiving tezacaftor/ivacaftor (TI). The remaining 30 patients were excluded because follow-up data were unavailable. Baseline pulmonary function assessments were performed on the day of treatment initiation. Follow-up assessments were generally conducted approximately 12 months later, although some were delayed because patients attended visits primarily to collect medication without undergoing a complete clinical evaluation. All follow-up assessments included in the analysis were performed within 12–24 months after treatment initiation. The flow of patients through the study is illustrated in Figure 1.

Pulmonary exacerbations requiring intravenous antibiotic treatment were assessed separately. Data were collected for the 24 months preceding CFTR modulator therapy initiation and the first 12 months after treatment initiation. Exacerbation rates were expressed as events per patient-year to account for the different observation periods.

The study protocol was approved by the Bioethics Committee of the Institute of Mother and Child in Warsaw, Poland (resolution no. 25/2025, issued on 4 April 2025).

### 4.2. Pulmonary Function Testing

Body plethysmography was performed using the MasterScreen BodyDiff system (CareFusion/Jaeger, Höchberg, Germany) in accordance with ATS/ERS technical standards [15,16]. The evaluated parameters included total airway resistance (Rtot), effective airway resistance (Reff), functional residual capacity (FRC), residual volume (RV), total lung capacity (TLC), and the RV/TLC ratio. Reference values for plethysmographic parameters were based on the equations of Zapletal et al. (1987) [27], as implemented in the measurement software, and were maintained throughout the study period. Results were expressed as percent predicted and/or z-scores.

For the categorical analyses, values exceeding 120% predicted were classified as elevated using study-defined thresholds. Parameter-specific z-score criteria were applied, as described in Section 4.6.

Measurement quality was initially assessed using the device’s automated quality-control functions. When necessary, the automated quality assessment was reviewed by the operator to verify technical acceptability. Only technically acceptable measurements were included in the analysis. Paired analyses were restricted to patients with valid measurements at both baseline and follow-up for each parameter.

Spirometry was performed using either a Vyntus IOS system (Vyaire Medical, Höchberg, Germany) or the spirometry module of the MasterScreen BodyDiff system (CareFusion/Jaeger, Germany). All measurements were obtained with patients in a seated position, in accordance with ATS/ERS technical standards [23]. At least three technically acceptable forced expiratory maneuvers were required. FEV1 and FVC were expressed as percent predicted values and z-scores using the Global Lung Function Initiative 2012 (GLI-2012) reference equations [29].

Multiple-breath nitrogen washout (MBNW) was performed using the Exhalyzer D system (Eco Medics AG, Duernten, Switzerland). All baseline and follow-up measurements were analyzed using Spiroware software version 3.3.1, which incorporates a correction for the previously identified cross-sensitivity error of the oxygen and carbon dioxide sensors. Ventilation inhomogeneity was assessed using the lung clearance index (LCI_2.5_), calculated as the cumulative expired volume divided by functional residual capacity (FRC) at the point when the end-tidal nitrogen concentration decreased to 2.5% of its initial value. Measurements were performed during tidal breathing under continuous operator supervision. Breathing patterns and potential leaks were monitored throughout each trial. Measurements affected by leaks or other technical abnormalities were terminated and repeated. At least three technically acceptable washout trials were required, with between-trial variability not exceeding 5% for LCI_2.5_ and 10% for FRC. Only measurements meeting the predefined quality and repeatability criteria were included in the analysis. The same measurement system and protocol were used throughout the study period. LCI_2.5_ values > 6.98 were classified as elevated, consistent with the threshold applied in our previous study [14].

### 4.3. Pulmonary Exacerbations

Clinical data on pulmonary exacerbations were extracted from electronic medical records. Pulmonary exacerbations were defined according to the criteria described by Fuchs et al. [30]. Only exacerbations requiring intravenous antibiotic treatment were included in the analysis.

Data were collected for two predefined observation periods: the 24 months preceding CFTR modulator therapy initiation and the first 12 months following treatment initiation. To account for the different lengths of these periods, exacerbation rates were annualized and expressed as the number of intravenous antibiotic-treated pulmonary exacerbations per patient-year.

### 4.4. Patient-Reported Outcomes

Health-related quality of life was assessed using the Cystic Fibrosis Questionnaire–Revised (CFQ-R) in 47 patients aged 12 years or older who completed questionnaires at baseline and follow-up (94 questionnaires in total). Of these, 42 patients had complete paired data across all eight analyzed CFQ-R domains and were included in the longitudinal analysis of domain scores. These patients also had paired pulmonary function data available for the exploratory correlation analyses.

### 4.5. Study Outcomes

The primary focus of the study was to assess longitudinal changes in plethysmographic parameters, including airway resistance (Rtot and Reff), functional residual capacity (FRC), residual volume (RV), total lung capacity (TLC), and the RV/TLC ratio. Secondary outcomes included changes in spirometric indices (FEV1 and FVC) and lung clearance index (LCI_2.5_). Exploratory analyses evaluated pulmonary exacerbations requiring intravenous antibiotics, health-related quality of life (CFQ-R), correlations between pulmonary function and patient-reported outcomes, and categorical changes in lung function parameters.

Given the retrospective observational design, this outcome hierarchy was established to clarify the analytical focus of the revised manuscript rather than representing prospectively prespecified endpoints.

During the preparation and revision of this manuscript, the authors used GPT-6 (GPT-6, OpenAI, San Francisco, CA, USA) primarily to assist with scientific and linguistic editing, improve text clarity and eliminate repetition. The tool was also used to support discussions of statistical methods; however, all statistical analyses were independently performed and verified by the authors using Statistica and R.

### 4.6. Statistical Analysis

Statistical analyses were performed using Statistica version 13.3 (TIBCO Software Inc., Palo Alto, CA, USA). R software (version 4.1.0; R Foundation for Statistical Computing, Vienna, Austria) was additionally used to estimate the confidence interval for the exploratory LCI_2.5_–CFQ-R correlation.

For each pulmonary function parameter, paired analyses included only patients with technically acceptable measurements at both baseline and follow-up. As data availability and technical acceptability varied across parameters, the number of complete paired observations differed between analyses and is reported in the corresponding tables.

The ETI and TI groups differed substantially in age, sex distribution, CFTR genotype, and sample size, reflecting differences in clinical eligibility for CFTR modulator treatment. These baseline differences, together with the unequal group sizes, limit the comparability of the treatment groups. Moreover, age-related growth and maturation may influence lung volume measurements in pediatric patients. As the study was designed to assess longitudinal changes within each treatment group rather than comparative treatment efficacy, no adjusted between-group analysis was performed. Therefore, differences in the magnitude or statistical significance of within-group changes should not be interpreted as evidence of superior efficacy of either treatment regimen. No direct between-group efficacy comparisons were performed.

Continuous pulmonary function outcomes are presented as mean ± standard deviation (SD) at baseline and follow-up. Within-patient changes were calculated as follow-up minus baseline values and are reported as mean change ± SD, together with 95% confidence intervals calculated using Student’s t-distribution. The Wilcoxon signed-rank test was used to assess paired changes. Exact *p*-values are reported whenever available, and the number of complete paired observations is provided for each parameter.

Categorical changes in pulmonary function parameters between baseline and follow-up were assessed using McNemar’s test with Yates’ continuity correction, with two-sided exact McNemar tests performed as a sensitivity analysis. Parameter-specific classification criteria were applied. FEV_1_ and FVC z-scores < −1.64 were classified as reduced, and LCI_2.5_ values > 6.98 as elevated. For Rtot, Reff, RV, TLC, and RV/TLC expressed as percent predicted, values > 120% were classified as elevated using study-defined thresholds. RV and TLC z-scores > +1.64 were classified as elevated, whereas RV/TLC z-scores outside the range of −1.64 to +1.64 were classified as abnormal. McNemar’s test assessed changes between these binary categories. Benjamini–Hochberg adjustment was applied separately within each treatment group and testing approach. Changes in CFQ-R domain scores were analyzed using complete paired observations (*n* = 42). Results are presented as mean ± standard deviation (SD), with mean within-patient changes calculated as follow-up minus baseline and reported with 95% confidence intervals. *p*-values were adjusted for multiple comparisons across the eight CFQ-R domains using the Benjamini–Hochberg procedure. The 95% confidence interval for the Spearman correlation between changes in LCI_2.5_ and CFQ-R respiratory domain scores was estimated using a nonparametric paired bootstrap procedure with 10,000 resamples and the bias-corrected and accelerated (BCa) method, implemented using the boot package in R version 4.1.0. The confidence interval was not adjusted for multiple comparisons.

Given the retrospective design, unequal group sizes, and multiple physiological outcomes assessed, the analyses were considered exploratory. Therefore, the results should be interpreted as hypothesis-generating rather than confirmatory.

## 5. Conclusions

ETI treatment was associated with significant changes in airway resistance, TLC, and the RV/TLC ratio. Although the reduction in RV/TLC suggests a favorable change in an index of relative air trapping, it should not be interpreted as direct evidence of reduced absolute trapped gas volume, particularly in the absence of a significant reduction in RV.

Plethysmography, particularly airway resistance and RV/TLC, may therefore provide complementary information when monitoring response to CFTR modulator therapy. Given the retrospective design and limited sample size, these findings should be considered exploratory and require confirmation in prospective studies.

## Figures and Tables

**Figure 1:**
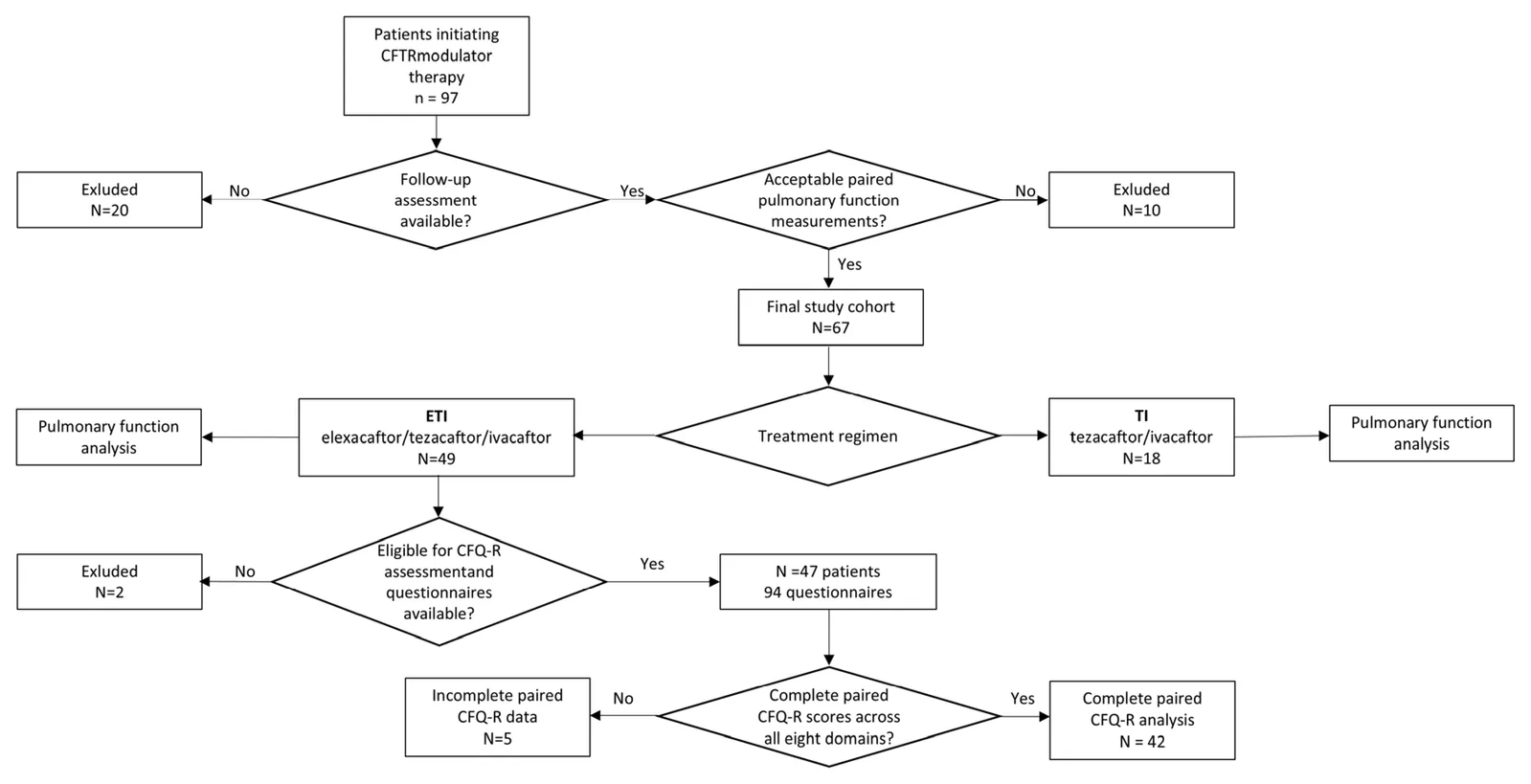
Study flowchart and patient selection.

**Table 1:** Baseline characteristics of patients according to CFTR modulator therapy.

Parameter	ETI	TI
Number of patients	49	18
Age at study initiation, years, mean ± SD	14.01 ± 1.73	9.72 ± 2.28
Female (%)	29 (59.2%)	7 (38.9%)
Male (%)	20 (40.8%)	11 (61.1%)
CFTR genotype category, *n* (%)		
F508del/F508del	25 (51.0%)	13 (72.2%)
F508del/other	24 (49.0%)	5 (27.8%)
IV antibiotic-treated pulmonary exacerbations before modulator therapy, mean ± SD events per patient-year	0.59 ± 0.82	0.75 ± 1.02

**Table 2:** Changes in pulmonary function parameters in the ETI-treated group.

Parameter	ETI Baseline	ETI Follow-Up	Mean Change ± SD	95%CI	*p* *	*p* Adjusted B-H	*N*
**Spirometry**							
FEV_1_, % predicted	91.53 ± 17.51	102.35 ± 17.38	10.82 ± 14.11	6.76; 14.87	<0.001	<0.001	49
FEV_1_ z-score	−0.70 ± 1.46	0.11 ± 1.16	0.81 ± 0.90	0.56; 1.07	<0.001	<0.001	49
FVC, % predicted	97.49 ± 14.40	100.73 ± 11.63	3.24 ± 7.67	1.04; 5.45	0.005	0.005	49
FVC z-score	−0.23 ± 1.23	0.06 ± 0.98	0.28 ± 0.68	0.09; 0.48	0.005	0.005	49
**Multiple-breath** **nitrogen** **washout**							
Lung clearance index (LCI_2.5_)	9.49 ± 2.91	7.53 ± 1.76	−1.96 ± 1.69	−2.44; −1.47	<0.001	<0.001	49
**Plethysmography**							
Total airway resistance (Rtot) % predicted	158.63 ± 61.49	139.51 ± 37.48	−19.12 ± 56.63	−35.39; −2.86	0.008	0.020	49
Effective airway resistance (Reff) % predicted	131.43 ± 60.23	109.12 ± 32.89	−22.31 ± 53.84	−37.77; −6.84	0.002	0.006	49
Functional residual capacity (FRC), % predicted	113.28 ± 18.84	111.55 ± 21.28	−1.73 ± 13.52	−5.62; 2.15	0.538	0.611	49
Functional residual capacity (FRC) z-score	0.99 ± 1.78	0.82 ± 2.04	−0.18 ± 1.22	−0.53; 0.17	0.619	0.619	49
Residual volume (RV), % predicted	119.36 ± 41.20	117.77 ± 40.17	−1.58 ± 29.98	−10.29; 7.12	0.424	0.606	48
Residual volume (RV) z-score	0.69 ± 1.97	0.66 ± 1.87	−0.029 ± 1.51	−0.47; 0.41	0.559	0.611	48
Total lung capacity (TLC), % predicted	103.75 ± 13.25	109.63 ± 13.23	5.88 ± 8.81	3.32; 8.43	<0.001	<0.001	4817
Total lung capacity (TLC) z-score	0.38 ± 1.82	1.14 ± 1.78	0.76 ± 1.10	0.45; 1.08	<0.001	<0.001	48
RV/TLC ratio, % predicted	113.87 ± 33.05	106.25 ± 28.55	−7.62 ± 24.00	−14.59; −0.67	0.018	0.030	48
RV/TLC ratio, z-score	0.84 ± 2.01	0.38 ± 1.69	−0.46 ± 1.45	−0.89; −0.04	0.017	0.030	48

Data are presented as mean ± standard deviation (SD). Mean changes were calculated as follow-up minus baseline, with 95% confidence intervals (CIs) estimated using Student’s t-distribution. *p*-values were obtained using the Wilcoxon signed-rank test and adjusted for multiple comparisons using the Benjamini–Hochberg procedure. Sample sizes (*n*) represent patients with complete paired measurements for each parameter. * *p*-values were obtained using the Wilcoxon signed-rank test.

**Table 3:** Changes in pulmonary function parameters in the TI-treated group.

Parametr	TI Baseline	TI Follow-up	Mean Change ± SD	95%CI	*p* *	*p* Adjusted B-H	*N*
**Spirometry**							
FEV_1_, % predicted	94.00 ± 13.32	89.64 ± 16.38	−4.35 ± 15.09	−12.11; 3.40	0.074	0.105	17
FEV_1_ z-score	−0.50 ± 1.10	−0.89 ± 1.40	−0.39 ± 1.31	−1.06; 0.28	0.084	0.105	17
FVC, % predicted	97.23 ± 9.80	91.29 ± 12.09	−5.94 ± 8.63	−10.38; −1.505	0.012	0.043	17
FVC z-score	−0.27 ± 0.85	−0.75 ± 1.04	−0.483 ± 0.75	−0.873; −0.093	0.017	0.043	17
**Multiple-breath** **nitrogen** **washout**							
Lung clearance index (LCI_2.5_)	7.87 ± 1.58	7.86 ± 1.67	−0.0006 ± 1.21	−0.624; 0.623	0.943	0.943	17
**Plethysmography**							
Total airway resistance (Rtot) % predicted	148.29 ± 39.50	154.00 ± 83.10	5.70 ± 93.18	−42.20; 53.61	0.831	0.981	17
Effective airway resistance (Reff) % predicted	108.52 ± 30.86	111.00 ± 68.90	2.47 ± 71.27	−34.17; 39.11	0.589	0.981	17
Functional residual capacity (FRC), % predicted	105.47 ± 22.87	104.88 ± 28.03	−0.59 ± 29.94	−15.98; 14.81	0.905	0.981	17
Functional residual capacity (FRC) z-score	0.28 ± 2.12	0.38 ± 2.80	0.10 ± 2.82	−1.35; 1.55	0.981	0.981	17
Residual volume (RV), % predicted	112.80 ± 44.86	107.00 ± 31.49	−5.80 ± 45.63	−31.07; 19.47	0.691	0.981	15
Residual volume (RV) z-score	0.32± 2.27	0.09 ± 1.67	−0.23 ± 2.29	−1.5; 1.04	0.842	0.981	15
Total lung capacity (TLC), % predicted	96.76 ± 16.13	92.65 ± 10.91	−4.12 ± 15.67	−12.18; 3.94	0.103	0.620	17
Total lung capacity (TLC) z-score	−0.69 ± 2.38	−1.13 ± 1.64	−0.44 ± 2.35	−1.65; 0.76	0.124	0.620	17
RV/TLC ratio, % predicted	109.13 ± 33.08	111.60 ± 30.00	2.47 ± 36.40	−17.69; 22.62	0.909	0.981	15
RV/TLC ratio, z-score	0.59 ± 2.15	0.70 ± 1.81	0.11 ± 2.36	−1.19; 1.42	0.233	0.777	15

Data are presented as mean ± standard deviation (SD). Mean changes were calculated as follow-up minus baseline, with 95% confidence intervals (CIs) estimated using Student’s t-distribution. *p*-values were obtained using the Wilcoxon signed-rank test and adjusted for multiple comparisons using the Benjamini–Hochberg procedure. Sample sizes (*n*) represent patients with complete paired measurements for each parameter. * *p*-values were obtained using the Wilcoxon signed-rank test.

**Table 4:** Number of patients exceeding predefined thresholds before and after CFTR modulator therapy.

Parameter	ETI Baseline	ETI Follow-Up	TI Baseline	TI Follow-Up
Rtot %pred. > 120%	16 (32.7%)	11 (22.4%)	13 (76.5%)	10 (58.8%)
Reff %pred. > 120%	10 (20.4%)	4 (8.2%)	6 (35.3%)	4 (23.5%)
RV% pred. > 120%	13 (27.1%)	10 (20.8%)	7 (46.7%)	7 (46.7%)
RV z-score > 1.64	7 (14.6%)	5 (10.4%)	2 (13.3%)	3 (20.0%)
TLC% pred. > 120%	4 (8.3%)	5 (10.4%)	1 (5.9%)	0 (0%)
TLC z-score > 1.64	6 (12.5%)	10 (20.8%)	4 (23.5%)	0 (0%)
RV/TLC %pred. > 120%	9 (18.8%)	7 (14.6%)	5 (33.3%)	6 (40.0%)
RV/TLC z-score > 1.64	7 (14.6%)	4 (8.3%)	4 (26.7%)	5 (33.3%)
FRC% pred. > 120%	10 (20.4%)	10 (20.4%)	2 (11.8%)	4 (23.5%)
FRC z-score > 1.64	9 (18.4%)	2 (4.1%)	2 (11.8%)	5 (29.5%)

Data are presented as *n* (%). The table presents the proportions of patients exceeding the specified parameter-dependent thresholds. Values > 120% predicted were classified as elevated for the purposes of this analysis, whereas z-scores > +1.64 were considered above the upper reference limit. These thresholds were used to describe the distribution of elevated values and should not be interpreted as universally validated criteria for physiological abnormalities across all parameters.

**Table 5:** Paired categorical analysis of lung function parameters.

Parameter	ETI Group McNemar χ^2^	ETI Group *p*-Value (Yates-Corrected)	ETI Group Exact *p*-Value	ETI Group BH-Adjusted Exact *p*-Value	TI Group McNemar χ^2^	TI Group *p*-Value (Yates-Corrected)	TI Group Exact *p*-Value	TI Group BH-Adjusted Exact *p*-Value
LCI_2.5_	14.45	<0.001	<0.001	<0.001	2.25	0.134	0.125	1.000
FEV1 z-score	8.10	0.004	0.002	0.011	1.50	0.220	0.219	1.000
FVC z-score	1.30	0.248	0.250	0.530	0.50	0.470	0.500	1.000
Reff %predicted	5.79	0.016	0.013	0.047	0.00	1.000	1.000	1.000
Rtot %predicted	0.27	0.607	0.607	0.835	0.25	0.617	0.625	1.000
RV %predicted	2.12	0.140	0.143	0.394	0.00	1.000	1.000	1.000
RV z-score	0.13	0.720	0.727	0.888	0.50	0.479	1.000	1.000
TLC z-score	0.00	1.000	1.00	1.00	1.13	0.289	0.289	1.000
TLC %predicted	0.25	0.617	1.00	1.00	-	-	1.000	1.000
RV/TLC %predicted	0.75	0.386	0.388	0.609	0.17	0.683	1.000	1.000
RV/TLC z-score	1.13	0.289	0.289	0.530	0.00	1.000	1.000	1.000

Categorical changes were assessed using McNemar’s test with Yates’ continuity correction and two-sided exact McNemar tests. Benjamini–Hochberg adjustment was applied separately by treatment group and testing approach. Reduced FEV_1_ and FVC were defined as z-scores < −1.64, and elevated LCI_2.5_ as >6.98. Study-defined thresholds of >120% predicted were applied to Rtot, Reff, RV, TLC, and RV/TLC. Elevated RV and TLC z-scores were defined as >+1.64, whereas RV/TLC z-scores outside ±1.64 were classified as abnormal. For TLC % predicted in the TI group, only the exact McNemar test result was reported because there was only one discordant pair.

**Table 6:** Changes in annualized pulmonary exacerbation rates before and after CFTR modulator therapy.

Parameter	ETI (*N* = 49)	TI (*N* = 18)
Before treatment, mean ± SD	0.59 ± 0.82	0.75 ± 1.02
After treatment, mean ± SD	0.16 ± 0.43	1.11 ± 2.08
Mean change (Δ) ± SD	−0.43 ± 0.78	+0.36 ± 1.25
Wilcoxon signed-rank test, *p*-value	<0.001	0.508
Decreased exacerbation rate, *n* (%)	24 (49.0%)	6 (33.3%)
Increased exacerbation rate, *n* (%)	2 (4.1%)	4 (22.2%)
Unchanged exacerbation rate, *n* (%)	23 (46.9%)	8 (44.4%)

Data are presented as mean ± standard deviation (SD) or *n* (%). Pulmonary exacerbation rates are expressed as intravenous antibiotic-treated events per patient-year, calculated over 24 months before and 12 months after treatment initiation. Mean change (Δ) was calculated as follow-up minus baseline. Within-group comparisons were performed using the Wilcoxon signed-rank test.

**Table 7:** Changes in CFQ-R domain scores between baseline and follow-up.

Parameter	*n*	Baseline, Mean ± SD	Baseline and Follow-Up (*n* = 42), Mean ± SD	Mean Change ± SD	95%CI	*p*	*p*-HB Adjusted
CFQ-R—Physical domain	42	89.55 ± 16.28	96.59 ± 6.53	7.04 ± 14.33	2.58; 11.51	0.001	0.004
CFQ-R—Emotional domain	42	84.48 ± 12.90	87.20 ± 11.65	2.72 ± 15.51	−2.11; 7.55	0.280	0.320
CFQ-R—Social functioning	42	71.05 ± 17.22	78.89 ± 15.60	7.84 ± 17.95	2.25; 13.44	0.016	0.032
CFQ-R—Body image	42	83.60 ± 19.36	83.33 ± 16.87	−0.26 ± 19.86	−6.45; 5.92	0.940	0.940
CFQ-R—Eating domain	42	85.71 ± 17.64	89.42 ± 16.08	3.70 ± 17.81	−1.85; 9.25	0.240	0.320
CFQ-R—Treatment domain	42	73.01 ± 15.93	77.78 ± 16.09	4.76 ± 15.74	−0.14; 9.67	0.050	0.080
CFQ-R—Respiratory domain	42	82.94 ± 16.32	95.50 ± 8.32	12.57 ± 16.11	7.55; 17.58	<0.001	<0.001
CFQ-R—Digestive domain	42	85.18 ± 17.64	92.06 ± 12.09	6.88 ± 16.63	1.69; 12.06	0.008	0.021

Data are presented as mean ± SD. All analyses included 42 patients with complete paired CFQ-R scores across the eight domains. Mean changes were calculated as follow-up minus baseline. The 95% confidence intervals refer to mean within-patient changes and were calculated using Student’s t-distribution. *p*-values were obtained using the Wilcoxon signed-rank test and adjusted for eight comparisons using the Benjamini–Hochberg procedure.

## Data Availability

The data presented in this study are available on request from the corresponding author due to privacy and ethical restrictions.

## Abbreviations

The following abbreviations are used in this manuscript:

BCa	bias-corrected and accelerated method
B-H	Benjamini–Hochberg procedure
CF	cystic fibrosis
CFQ-R	Cystic Fibrosis Questionnaire-Revised
CFTR	cystic fibrosis transmembrane conductance regulator
ETI	elexacaftor/tezacaftor/ivacaftor
FEV1	forced expiratory volume in 1 s
FEV1%predicted	percent predicted forced expiratory volume in 1 s
FRC	functional residual capacity
FVC	forced vital capacity
IV	intravenous
LCI_2.5_	lung clearance index at 2.5% of the starting tracer gas concentration
MBNW	multiple-breath nitrogen washout
mCFTR	CFTR modulator therapy
MRI	magnetic resonance imaging
Reff	effective airway resistance
Rtot	total airway resistance
RV	residual volume
RV/TLC	residual volume-to-total lung capacity ratio
SD	standard deviation
TI	tezacaftor/ivacaftor
TLC	total lung capacity
